# The Address in Surgery

**Published:** 1900-08-04

**Authors:** Frederick Treves

**Affiliations:** Surgeon-Extraordinary to H.M. the Queen; Surgeon-in-Ordinary to H.R.H. the Duke of York; Consulting Surgeon to the London Hospital.


### THE ADDRESS IN SURGERY.

By Frederick Treves, F.R.C.S., Surgeon-Extraordinary to H.M. the Queen; Surgeon-in-Ordinary to H.R.H. the Duke of York; Consulting Surgeon to the London Hospital.

Many who went to hear Mr. Treves would no doubt be disappointed to find that on the particular subject in regard to which his name has lately been so prominently brought before the public he was silent, and that he neither made South Africa his theme, nor devoted himself to a consideration of any of those great surgical problems on which the experience of the war is imagined to have thrown so much new light. Mr. Treves occupied himself principally in a consideration of the progress made by the surgeon during the century, and in so doing he began by drawing a picture of the social life of England a hundred years ago. A sympathetic literature has, he said, left us ample records of the men and women of the time and of the scenes amid which they moved, records rich with the littleness of personal affairs. The life of men was then largely spent in the country, in sleepy villages, and in sober, self-esteeming country towns. Travelling was a luxury limited to the few. The many were content to live and die within sight of fields and spires which had been familiar from babyhood. These simple folk were comparatively free from the fever of social ambition, from the almost savage conflict of modern commerce, and from the bewildering hurry of events which compass the peace of later times. London was then not one-fourth of its present size; the great City ended, towards the west, at Sloane Street, and beyond were quiet fields and meadows intersected by footpaths, certain of which led to the hamlet of Kensington, and the little riverside village of Chelsea. No railway terminus flaunted its hideous structure in the City's midst, and neither omnibus nor cab nor tram car invaded the narrow streets. There was no telegraph and no telephone. The postal system was without form and void. The gayest streets were lit by oil lamps, and the gloom by night was only equalled by the dirt by day. As to the surgeon in those days, he was but a sorry element in social life. In the great towns and cities there were esteemed practitioners of surgery who were eminent by reason of their scientific work and their successful practice, but their numbers were few. The surgeon or common practitioner in the village and town in these early days has been depicted by many writers. We find him an ignorant, illiterate, sordid creature, not above the allurements of money-grubbing, and not without suspicion of dishonest practices and of a leaning towards the bottle. He was to a large extent a mere retailer of physic, and in the public eye he ranked with the quacks and nostrum-sellers with whom he competed. Mr. Treves then proceeded to describe the immensity of the change which has taken place in the position occupied by the surgeon, and gave a sketch of the various influences which have co-operated to this result, treating the subject under the three heads: (1) The surgeon as an adviser; (2) the surgeon as a man of learning; and (3) the surgeon as an operator. Under the latter heading he said that during the nineteenth century the surgeon as an operator had passed through a rapid metamorphosis, and had now reached at least the level of the unexpected. It might have been supposed that there was little to be learnt in the way of using a knife which had not been learnt during a period of over eighteen hundred years, for there were surgeons before the Christian era, and that twenty centuries would have exhausted the methods by which a limb could be cut off. Yet in 1800 modes of amputation were in vogue which are now regarded as uncouth, while in regard to the surroundings in which surgery is performed, since the days when the surgeon stepped into the arena of the operating theatre "as a matador strides into the ring," having around him "a gaping audience and before him a conscious victim, quivering, terror-stricken, and palsied with expectation," the change has been enormous. Turning to the circumstances which have conduced more especially to this change, he said that prominent among them were: (1) An improved knowledge of anatomy. (2) A readier method of arresting hæmorrhage, more especially by the use of pressure forceps, which, he said, represent the most valuable addition which has ever been made to the surgeon's appliances. "No instrument has brought with it so great an assurance of security, or has done more to extend the area of safe operations." (3) The employment of anæsthetics. (4) The introduction of antiseptic measures. "When the century was young the touch of the operator was the touch of a tainted hand; the balm he poured into the wound was poisoned, and he himself undid the good his science strained to effect. . . . The change has been great, and its greatness lies in its littleness, for it is bound up with no more than this—that the surgeon has learnt to be clean." Turning now from the past, and from a survey of changes which have taken place, Mr. Treves fell into prophetic vein, and descanted on the surgeon of the future. While in days gone by the greater deeds of surgery were limited to cities and large towns, and the general practitioner seldom took up the scalpel except in minor necessities, we find at the close of the nineteenth century, he said, that the disposition of affairs is wholly altered. Where there was one surgeon there are now ten, and throughout the breadth of the land and to its utmost limits the work of the operator has extended. The more ambitious performances of surgery are no longer restricted to great centres, but are carried out in the little town, in the cottage hospital, and even in the cottage itself. The days of the great operator, of the one man to whom all came who could, are rapidly passing away. Indeed, the practice of pure surgery, which was at one time limited to the prominent few, is now becoming common to the many. In every great change there must—at first, at least—be some undesirable developments, and it is impossible to deny that the wider distribution of the practice of operative surgery may lead to the occasional performance of major operations by men who are not justified, either by experience or by training, in undertaking them. Furthermore there is, among the signs of the times, some evidence that the reaction in the matter of operative surgery is to some degree extreme, and that we are in danger of passing from the policy of doing too little to the policy of doing too much. Operations we know were too few in the past, but there is some foundation for the impression that they are occasionally too frequent in the present. One other matter which looms out of the future will suffice to bring this subject to a close. So many have been the artificial aids to clinical investigation which recent science has introduced that it comes to be a question whether the natural acumen of the surgeon will not deteriorate in proportion as he fails to encourage that particular learning which clings to the finger tips of all great diagnosticians. That there will be such a decadence is beyond doubt. Examples of the directions in which this loss will be felt come readily to mind. A considerable amount of skill, for example, is demanded in the examination of complex fractures, of lesions of deep-seated bones, and of injuries about joints. What was to be learnt of these troubles had to be acquired by a tedious manipulation demanding considerable refinement. The surgeon can now afford to dispense with a prolix examination, and can at once submit the inquiry to a demonstrator of the Roentgen rays. An obscure tumour—to take another instance—presents itself, and no longer is the surgeon compelled to trust to the acuteness of his inquiry and his patient review of all the physical details of the mass, for he can turn to the findings of the exploratory incision, the trochar, and the aspirator. Here once more an advantage is minimised by a loss. Or, again, the nature of an abdominal swelling is at once decided by an exploratory laparotomy. By such little operation a great advantage is gained, but an opportunity to add to one of the most refined forms of learning is lost, and it is impossible not to notice a tendency to resort too readily to this means of solution. The same in regard to the decision of the microscopist and the bacteriologist—useful, but attended with a loss. Those, therefore, who are concerned with the education of the surgeon of the future would do well to still cherish this ancient power, and to foster a memory of the fact that surgery is, in its very essence, a handicraft, and that in all that he does the surgeon's great endeavour should be to make his own hands self-sufficing.

